# Supplementary material for: Genetic Parameter and Hyper-Parameter Estimation Underlie Nitrogen Use Efficiency in Bread Wheat
Source: Int J Mol Sci. 2023 Sep 19;24(18):14275. doi: 10.3390/ijms241814275 (PMC10531695; doi:10.3390/ijms241814275)
Supplement: Supplementary file 1 [file ijms-24-14275-s001.zip › ijms-2574711-supplementary.pdf]

International Journal of *Molecular Sciences*

MDPI

Supplementary Figures to manuscript ijms-2574711-revised.

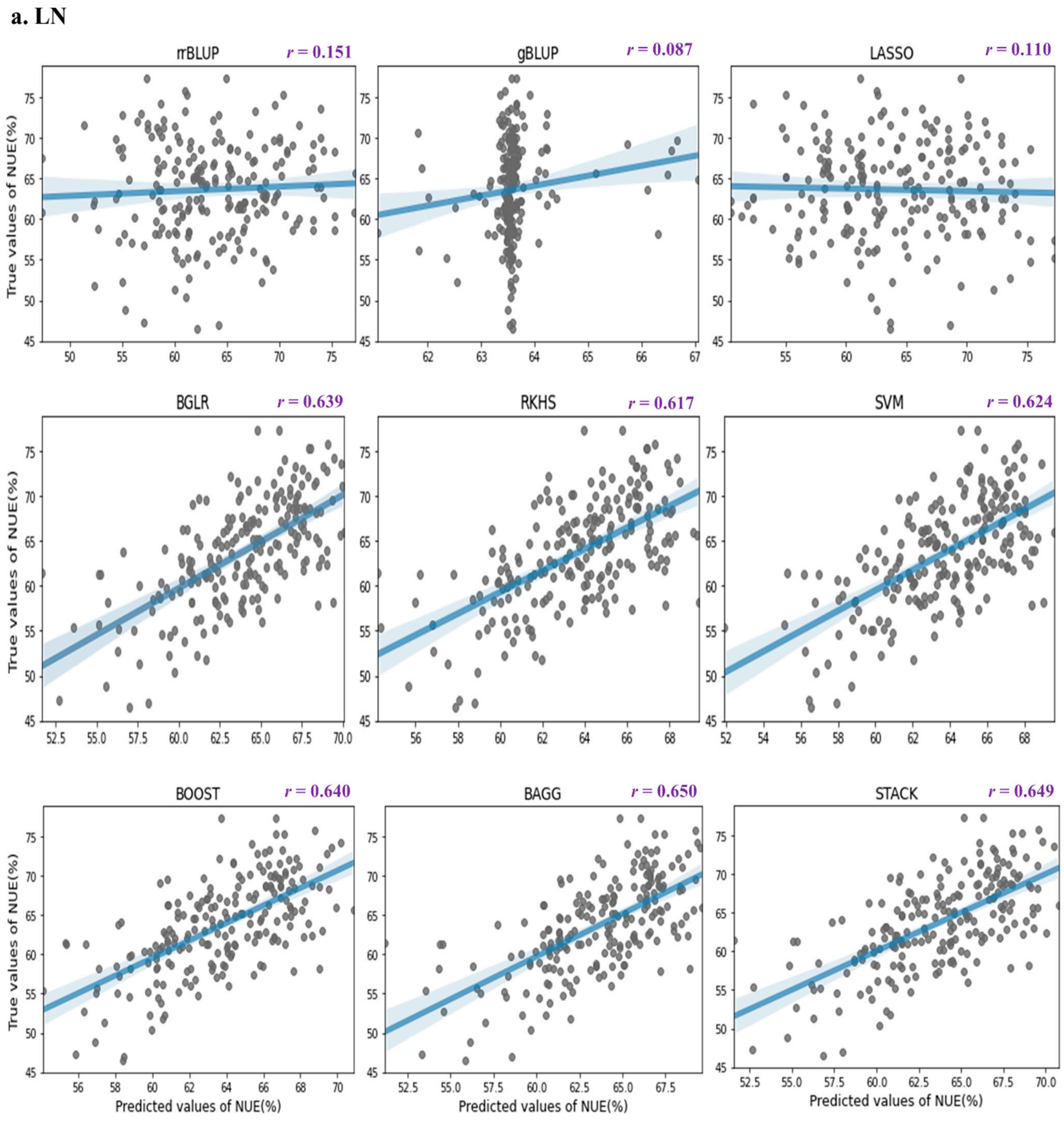

Figure S1. a. Regression plots of predicted values of NUE (%) versus true values of NUE (%) using different genomic selection models. To determine the accuracy of each model, the Pearson correlation was calculated for each model and is shown in purple. The gBLUP model and the BAGG model showed the lowest and highest correlation at low N levels, respectively.

b. HN

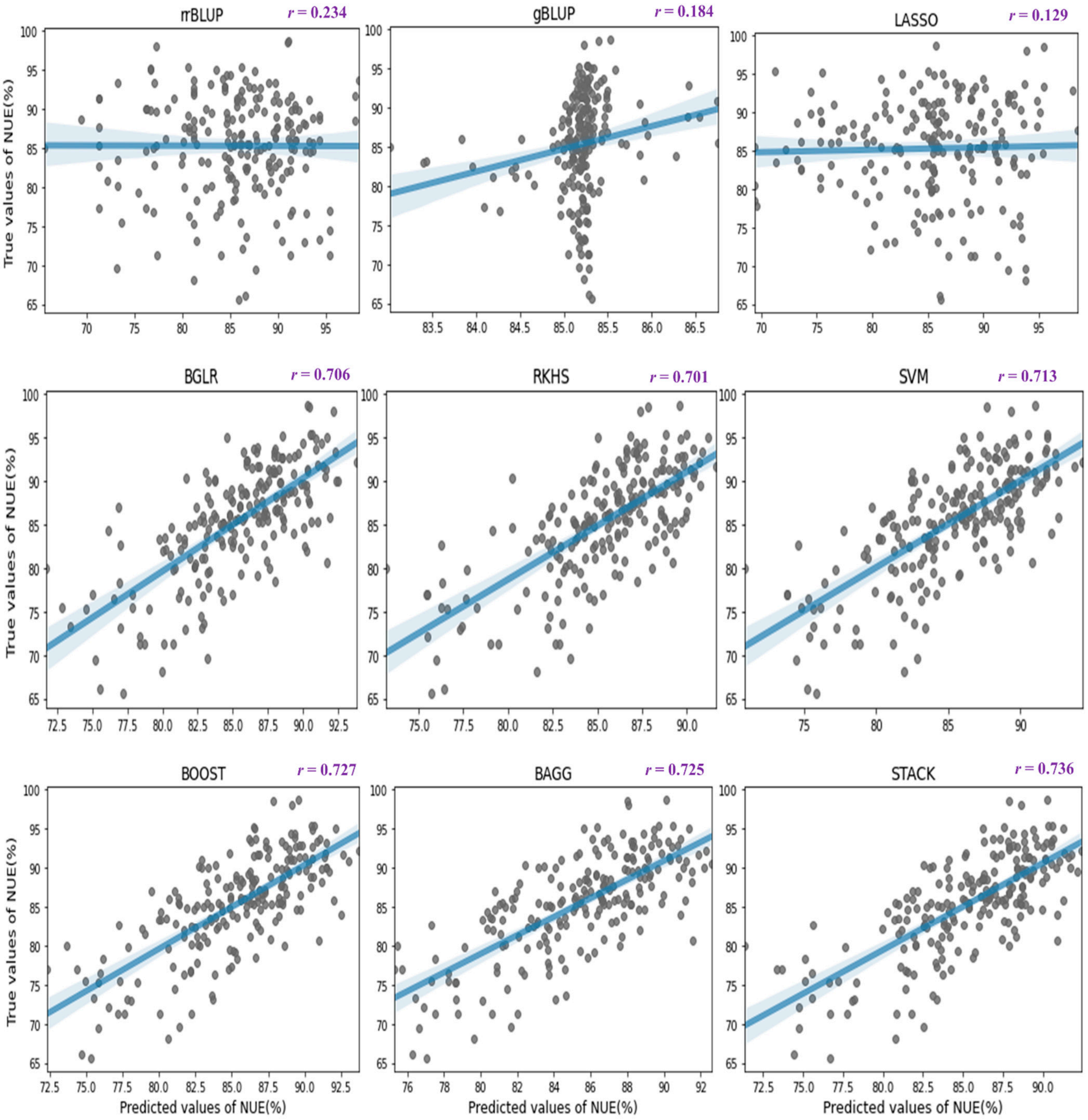

Figure S1. b. Regression plots of predicted values of NUE (%) versus true values of NUE (%) using different genomic selection models. To determine the accuracy of each model, the Pearson correlation was calculated for each model and is shown in purple. The LASSO model and the STACK model showed the lowest and highest correlation at high N levels, respectively.

Package description:

R/rrBLUP: Software for genomic prediction with the RR-BLUP mixed model (Endelman 2011, <doi:10.3835/plantgenome2011.08.0024>). One application is to estimate marker effects by ridge regression; alternatively, BLUPs can be calculated based on an additive relationship matrix or a Gaussian kernel.

R/BGLR: Bayesian Generalized Linear Regression. Please use the canonical form https://CRAN.R-project.org/package=BGLR to link to this page.

R/glmnet: Extremely efficient procedures for fitting the entire lasso or elastic-net regularization path for linear regression, logistic and multinomial regression models, Poisson regression, Cox model, multiple-response Gaussian, and the grouped multinomial regression; see <doi:10.18637/jss.v033.i01> and <doi:10.18637/jss.v039.i05>. There are two new and important additions. The family argument can be a GLM family object, which opens the door to any programmed family (<doi:10.18637/jss.v106.i01>). This comes with a modest computational cost, so when the built-in families suffice, they should be used instead. The other novelty is the relax option, which refits each of the active sets in the path unpenalized. The algorithm uses cyclical coordinate descent in a path-wise fashion, as described in the papers cited.

R/BWGS: Package for Breed Wheat Genomic Selection Pipeline. The R package 'BWGS' is developed by Louis Gautier Tran <louis.gautier.tran@gmail.com> and Gilles Charmet <gilles.charmet@inra.fr>. This repository is forked from original repository <https://forgemia.inra.fr/umr-gdec/bwgs> and modified as a R package.

R/kernlab: Kernel-based machine learning methods for classification, regression, clustering, novelty detection, quantile regression and dimensionality reduction. Among other methods 'kernlab' includes Support Vector Machines, Spectral Clustering, Kernel PCA, Gaussian Processes and a QP solver.

R/gbm: An implementation of extensions to Freund and Schapire's AdaBoost algorithm and Friedman's gradient boosting machine. Includes regression methods for least squares, absolute loss, t-distribution loss, quantile regression, logistic, multinomial logistic, Poisson, Cox proportional hazards partial likelihood, AdaBoost exponential loss, Huberized hinge loss, and Learning to Rank measures (LambdaMart). Originally developed by Greg Ridgeway.

R/ipred: Improved predictive models by indirect classification and bagging for classification, regression and survival problems as well as resampling based estimators of prediction error.
